# Familiarity is not notoriety: phenomenological accounts of face recognition

**DOI:** 10.3389/fnhum.2014.00672

**Published:** 2014-09-01

**Authors:** Davide Liccione, Sara Moruzzi, Federica Rossi, Alessia Manganaro, Marco Porta, Nahumi Nugrahaningsih, Valentina Caserio, Nicola Allegri

**Affiliations:** ^1^Lombard School of PsychotherapyPavia, Italy; ^2^Department of Brain and Behavioral Sciences, University of PaviaPavia, Italy; ^3^Nursing Home De RodolfiVigevano, Pavia, Italy; ^4^Department of Engineering, University of PaviaPavia, Italy

**Keywords:** face recognition, familiarity, inversion, facial expression, person, phenomenology

## Abstract

From a phenomenological perspective, faces are perceived differently from objects as their perception always involves the possibility of a relational engagement (Bredlau, 2011). This is especially true for familiar faces, i.e., faces of people with a history of real relational engagements. Similarly, valence of emotional expressions assumes a key role, as they define the sense and direction of this engagement. Following these premises, the aim of the present study is to demonstrate that face recognition is facilitated by at least two variables, familiarity and emotional expression, and that perception of familiar faces is not influenced by orientation. In order to verify this hypothesis, we implemented a 3 × 3 × 2 factorial design, showing 17 healthy subjects three type of faces (unfamiliar, personally familiar, famous) characterized by three different emotional expressions (happy, hungry/sad, neutral) and in two different orientation (upright vs. inverted). We showed every subject a total of 180 faces with the instructions to give a familiarity judgment. Reaction times (RTs) were recorded and we found that the recognition of a face is facilitated by personal familiarity and emotional expression, and that this process is otherwise independent from a cognitive elaboration of stimuli and remains stable despite orientation. These results highlight the need to make a distinction between famous and personally familiar faces when studying face perception and to consider its historical aspects from a phenomenological point of view.

## Introduction

Face recognition is an essential task for human daily life as it allows the identification of the person in front of you and provides the possibility of a relational engagement (Kleinke, 1986). Several types of information can be extracted from the perception of a face, ranging from age, gender and emotional states, but above all, identity (Morrison et al., 2001; Jenkins and Burton, 2008). Faces constitute a separate perceptual category, differing in many aspects from other stimuli, such as objects (Tanaka and Sengco, 1997). They are perceived holistically, in contrast with other objects, which receive an elaboration based on the processing of constitutive details (Tanaka and Sengco, 1997; Farah et al., 1998; Ge et al., 2006). Face perception is defined as “holistic” because faces are processed as gestalts, with single facial features (nose, mouth, eyes and so on) having a less fundamental role in respect to the global face configuration (Maurer et al., 2002). The result is that any kind of experimental manipulation preventing this kind of elaboration could result in an impairment in making a judgment about face identity. The most studied of these manipulations is the so called “face inversion effect”. This mechanism prevents the possibility to encode spatial information and causes the inability to perceive individual faces as a whole, forcing stimulus processing based on a system of specific and integrated features. This usually results in lower accuracy and slower reaction times (RTs; Valentine, 1988). Some interesting findings have been found in presenting inverted faces to patients with prosopagnosia, a neurological disorder characterized by the inability to recognize faces (Bauer, 1984; Grüter et al., 2008; Gainotti, 2014). Patients with congenital (Rivolta et al., 2012) and acquired prosopagnosia (Busigny and Rossion, 2010), show not to have holistic perceptual processing abilities, being minimally (if at all) affected by face inversion. Furthermore, some studies show better performance for inverted than upright faces, though this latter effect is not very common in either form of prosopagnosia (Farah et al., 1995a; Behrmann et al., 2005; Busigny and Rossion, 2010).

Besides the perceptual aspects of face recognition, great interest has been shown in the study of the elaboration of the so called “emotional valence” (Bruce and Young, 1986). Traditional cognitive models of face recognition speculate that facial identity and facial expressions are processed through different routes. Bruce and Young (1986), hypothesized the existence of two distinct elaboration pathways: one involved in identity recognition, the other in the analysis of facial expressions. The model is supported by clinical (Young et al., 1993), neurophysiological (Hasselmo et al., 1989) and neuroradiological (Winston et al., 2004) evidence, leading also to the formulation of a distributed neural system of face recognition (Haxby et al., 2000; Rivolta et al., 2014). However, there are some experimental evidences that undermine the dual route hypothesis. Many studies have shown an influence of facial expressions on identity recognition of newly learned faces (Foa et al., 2000; D’Argembeau and Van der Linden, 2007) and of famous faces (Gallegos and Tranel, 2005). Moreover, Van de Stock demonstrated that face identity perception mechanisms interact not only with the processing of facial expressions but also with bodily expressions (Van de Stock and de Gelder, 2014).

Some attention has also been concentrated on the study of emotional recognition in inverted faces. Literature on this topic is quite heterogeneous: while some studies found a detrimental effect of inversion only for the recognition of some emotional expressions (McKelvie, 1995; Calvo and Nummenmaa, 2008), some others found a general difficulty in recognizing inverted expressions for all types of emotions (Goren and Wilson, 2006). However, the most acknowledged idea is that the only expression not affected by inversion is happiness (Leppänen and Hietanen, 2004; Bombari et al., 2013). A limit of the above presented studies is that a distinction has not been made between famous and personally familiar faces, since the recognition of these two types of stimuli may differ in various aspects. In this regard, Herzmann et al. (2004), studied RTs, priming, and skin conductance response to unfamiliar, famous and personally familiar faces. They found faster RTs for both famous and personally familiar faces, but a greater skin conductance only for this last category. Moreover, recognition of personally familiar and famous faces seems to be based in different neurological areas. Taylor et al. (2009), in an fMRI study, compared unknown, famous and familiar faces, finding that the extent and areas of activation varied according to face type.

The three types of stimuli appear to be profoundly different if considered from a phenomenological perspective. Phenomenological theories claim that perception is an active process, structurally embodied, embedded, extended and enactive,^1^ and that person recognition is different from object recognition.

What we perceive is determined by what we can do, and this is valid for both objects and people (Noë, 2004): the difference is that while an object reveals itself in a pattern of possibilities of action, a face reveals itself in a pattern of relational possibilities. In fact, in encountering another person the most pressing task is relational engagement. In these terms it appears clear why familiar faces are different from famous and unknown faces: if we encounter a familiar person (i.e., a person who has a history of real relational engagements with us) many ways of being in engagement become vivid and start to pertain to our personal experience and to its significance. In observing a familiar person we experience ourselves in our personal possibilities of relational engagement. In this way, particular importance is given to the processing of emotional expressions, because they define the “sense”^2^ of this engagement (Bredlau, 2011). We therefore hypothesize that familiarity is a so powerful constituent of face perception to overcome the effect of the inversion of the stimulus and to be not influenced by emotional expressions.

Therefore, the aim of the present study is to investigate whether manipulations of orientation and expressions can influence the processing of facial identity of unfamiliar, personally familiar and famous faces. Our hypothesis is that inversion does not affect the vivid experiential perception of a familiar face, leading to similar RTs for inverted, compared to upright familiar faces. For this purpose, we presented our subjects with pictures of unfamiliar, famous and personally familiar faces, both upright and inverted, with three different emotional expressions: happy, neutral and sad/angry. The main element of evaluation was the RTs of our subjects during a face recognition task.

## Methods

### Participants

Seventeen adults (5 male; 12 female), with normal or correct-to-normal vision, ranging in age from 23 to 36 (*M* = 27.7, DS = 2.43 years), participated in this study. All participants were unaware of the purpose of the experiment. The study conformed to the national guidelines and regulations of the A.I.P. (Italian Association of Psychology), and was approved by the Lombard School of Psychotherapy ethical review committee. All subjects gave informed consent.

### Stimuli

Visual stimuli consisted of digitalized grayscale images of familiar, famous and unknown faces, displaying positive, negative or neutral expressions. All images were selected for high-resolution frontal views and forward eye-gaze. Pictures were homogenized for average brightness and contrast, and did not show significant differences in these parameters across categories. In accordance with the purpose of this study we avoided removing hair, glasses or other distinctive features from the portraits, in order to keep an authentic approach to face perception.

*Familiar faces*. These highly familiar faces consisted of pictures of 10 familiar people for each subject. The choice of familiar people was based on a questionnaire previously filled by the participants, which were asked to indicate 10 relatives or significant others (e.g., spouse, partner, etc.). The researchers contacted each familiar person and photographed them with three different expressions (positive, negative and neutral) making a total of 30 photos. Originally relatives were asked to pose happy, angry and neutral expressions. Nevertheless, due to subjects’ difficulty in reproducing intentionally a unequivocal angry faces we chose to categorize those facial expressions (and therefore also the others) on emotional valence (positive, negative and neutral) rather than on discrete emotional states. So, our negative familiar stimuli can encompass both angry and sad faces. Difficulty in producing negative expressions on command is shown in other studies (Öhman et al., 2001). All familiar people gave informed consent.

*Famous faces*. Famous people were selected for use in this experiment on the basis of findings from a pilot study. Sixteen celebrities, appearing regularly in the media (politicians, actors, television celebrities etc.), were chosen: three images for each celebrity, judged by the authors as having neutral, positive and negative expressions were downloaded from the Internet. Fifty-one subjects (32 female, 19 male), outside the study, were asked to rate portraits for notoriety and emotional expressions (as positive, negative or neutral). For each face, participants were asked to answer the question: “What’s the name of this person?” and to rate their notoriety on a Likert-type scale ranging from 0 (not at all familiar) to 6 (very familiar). To assess emotional expressions, participants were asked to judge if the expressions were positive, negative or neutral. The final stimulus set comprised 10 (5 male and 5 female) of the 16 celebrities who met the following criteria: identity recognized by 100% of participants and each emotional expression correctly rated by 85% of participants.

*Unknown faces*. 10 unknown faces were included in the experiment (for each participant we used photos of relatives and the significant others of other subjects).

**Figure 1:**
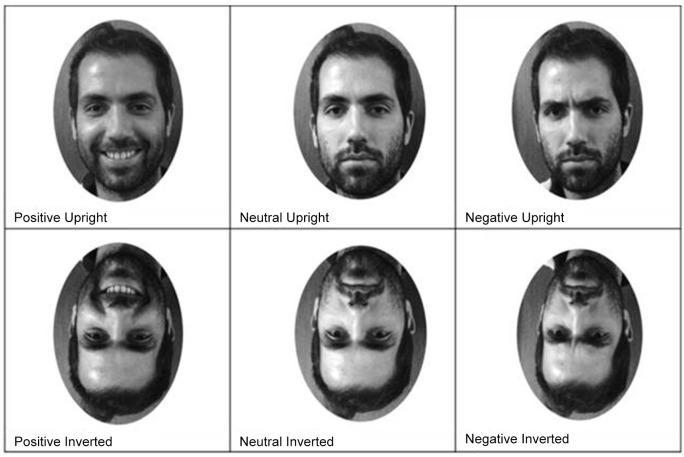
**Example of stimuli images**.

In total, 90 stimuli were used of 10 personally familiar, 10 famous and 10 faces unknown to the participants. Each face displayed the three expressions, and each stimuli were presented upright and inverted, for a total of 180 pictures (see Figure 1 for an example) and presented in a single session.

### Procedure

**Figure 2:**
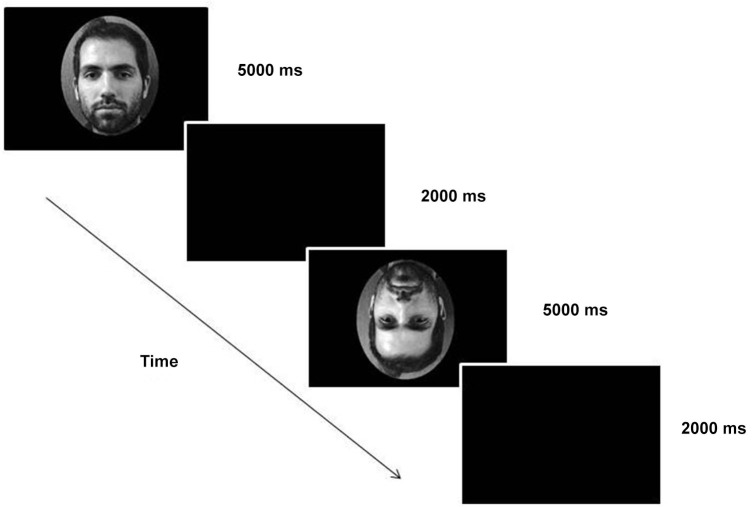
**Example of experiment**.

As participants arrived at the laboratory, they read the information sheet, completed the consent form and were informed that they would perform computer-based tasks. Participants were seated in a quiet room, approximately 60 cm from the screen, and viewed all 180 images in one continuous block. All images were presented once for 5000 ms in randomized order with a black inter-stimulus slide lasting 2000 ms (Figure 2). Participants were instructed to press, as quickly as possible, one of two keys (B and M-counterbalanced response across subjects) in agreement with subjective recognition judgment (whether the face was known or not). No training was given to the participants prior to the facial recognition task.

### Reaction time

Participants RTs were recorded by the Tobii Studio 1750 eye tracker software. Raw data of RTs were exported from Tobii Studio and processed using an *ad-hoc* software module developed with Microsoft Access. The obtained results were then adapted to SPSS databases in order to further explore the data through statistical analyses.

### Statistics

Data analyses were performed using SPSS Statistics for Windows.

Statistical analysis was performed on the logarithmic transformed data of RTs. The main purpose of this log transformation is to get the sampled data in line with the assumptions of parametric statistics (such as ANOVA) and to deal with outliers. A 3 (class: familiar/famous/unknown) × 3 (expression: positive/negative/neutral) × 2 (orientation: upright/inverted) repeated measures ANOVA explored whether RTs differed per stimuli. Stimulus type (familiar, famous and unknown), expressions (positive, negative and neutral) and orientation (upright and inverted) were entered as within-subjects variables. Effect sizes (partial eta-squared, $\eta_{p}^{2}$, for *F*-statistics) are reported together with *p*-values for significant main effects and interactions, and *post-hoc* *t*-tests were Bonferroni-corrected to require a significance value of *p* < 0.01. An $\eta_{p}^{2}$ value above 0.01 indicates a small effect, a $\eta_{p}^{2}$ above 0.06 a medium effect, and a $\eta_{p}^{2}$ above 0.14 a large effect. We used Mauchly’s Test of Sphericity to test the assumption of sphericity, if this assumption is violated, the *F*-statistic is positively biased rendering it invalid and increasing the risk of a Type I error. To overcome this problem, Greenhouse-Geisser correction was applied to the degrees of freedom (*df*).

## Results

Table 1 shows mean values of reaction times.

**Table 1:** **Means and standard error for reaction times**.

Orientation	Class	Expression	Mean	Std. error
Upright	Familiar	Positive	775.91	21.20
		Neutral	808.01	18.14
		Negative	729.13	17.41
	Famous	Positive	825.49	26.50
		Neutral	834.79	27.53
		Negative	822.16	25.94
	Unknown	Positive	822.83	10.32
		Neutral	827.08	13.45
		Negative	856.63	16.43
Inverted	Familiar	Positive	764.48	21.23
		Neutral	811.35	30.37
		Negative	757.09	23.37
	Famous	Positive	978.05	36.06
		Neutral	987.24	37.12
		Negative	1057.05	46.32
	Unknown	Positive	1016.90	27.41
		Neutral	1105.05	30.09
		Negative	1165.89	40.13

**Figure 3:**
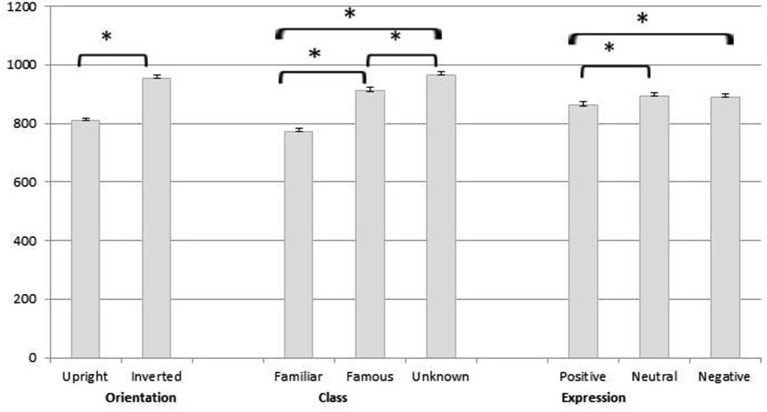
**Pairwise comparisons of the main effects of orientation, class and expression**. Single asterisk indicates significance at *p* < 0.001.

This analysis revealed the main effects of Orientation [*F*_(1,169)_ = 167.04, *p* < 0.001, $\eta_{p}^{2}$ = 0.50], Class [*F*_(1.87,315.39)_ = 69.80, *p* < 0.001, $\eta_{p}^{2}$ = 0.29] and Expression [*F*_(2,338)_ = 5.62, *p* = 0.004, $\eta_{p}^{2}$ = 0.03]. Pairwise comparisons (Figure 3) reveal that RTs in upright condition were lower than in inverted condition (*p* < 0.001); RTs in detecting familiar faces were significantly faster compared to both famous (*p* < 0.001) and unknown faces (*p* < 0.001). RTs were faster for famous compared to unknown (*p* = 0.001); and for positive compared to neutral (*p* = 0.01) and negative expressions (*p* = 0.01). No differences were found between neutral and negative expressions (*p* > 0.05). Analysis revealed that all two-way interactions were significant (Orientation × Expression [*F*_(2,338)_ = 11.16, *p* < 0.001, $\eta_{p}^{2}$ = 0.06]; Orientation × Class [*F*_(1.74,293.76)_ = 46.48, *p* < 0.001, $\eta_{p}^{2}$ = 0.21]; Class × Expression [*F*_(3.72,628.58)_ = 8.20, *p* < 0.001, $\eta_{p}^{2}$ = 0.05]).

**Figure 4:**
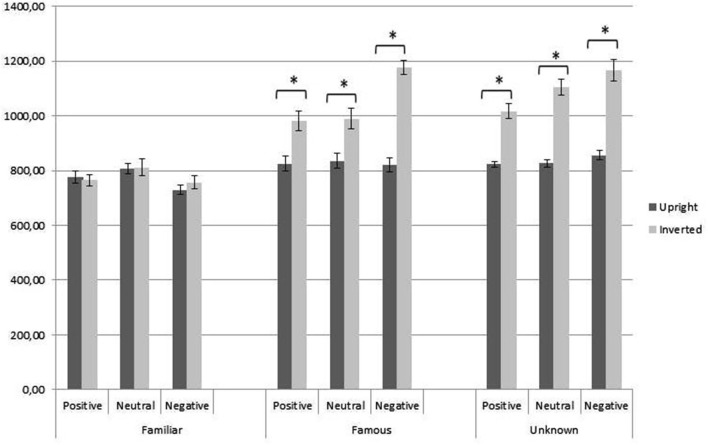
**Interaction between orientation, class and expression comparing for orientation**. Single asterisk indicates significance at *p* < 0.001.

We found a significant three way interaction between orientation, class and expression [*F*_(3.64,614.61)_ = 2.81, *p* = 0.02, $\eta_{p}^{2}$ = 0.02]. Interaction between orientation, class and expression comparing for orientation (Figure 4) showed no significant differences for familiar faces in RTs between upright and inverted condition for all the expressions; in famous and unknown categories, instead, RTs were significantly higher for inverted orientation for all the expressions.

**Figure 5:**
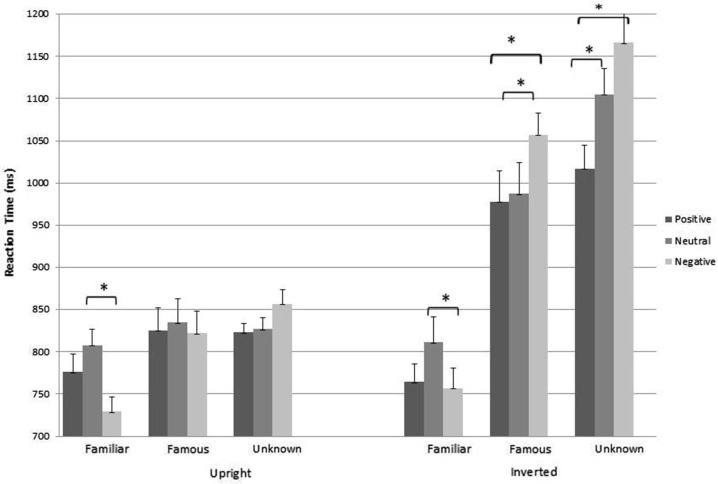
**Interaction between orientation, class and expression comparing for expression**. Single asterisk indicates significance at *p* < 0.001.

Interaction between orientation, class and expression, comparing for expression in upright condition (Figure 5, left part), showed that just in familiar we found a significant difference between neutral and negative expressions.

Interaction between orientation, class and expression comparing for expression in inverted condition (Figure 5, right part) showed that in familiar faces we replicate results of upright condition, in famous faces we found significant differences between negative and both positive and neutral, in unknown faces between positive and both neutral and negative.

## Discussion

The purpose of our study was to evaluate, in a face recognition task, the effects of different levels of face-familiarity (personally familiar, famous and unknown faces), orientation (upright or inverted) and emotional expressions (positive, neutral or negative). The main results can be summarized as follows: (1) regardless of orientation and expression, familiar faces are recognized faster than other stimuli; (2) inverted orientation does not seem to delay response times only for familiar faces; and (3) there appears to be a significant relation between familiarity and expression which is in turn affected by orientation.

Concerning the first issue, although based on RTs, our data are consistent with studies that show different psychophysiological responses for famous than unknown persons (Tranel et al., 1985; Ellis et al., 1999). In our study, faces of personally familiar people (relatives, spouse, partner, etc.) are identified more quickly compared to famous and unknown faces across all conditions. These data are consistent with Herzmann et al. (2004) who found higher autonomic responses for familiar, compared to both famous and unknown faces, although RTs did not differ between familiar and famous stimuli. This discordance can be explained by the different operative definitions of familiarity: in their study, Herzmann et al. (2004) used a broad concept of familiarity (portraits of lecturing staff), while we use a strict notion of familiarity and this difference may result in different RTs during the recognition task.

The second argument looks at the interaction between orientation and class, showing results that are consistent with studies that demonstrate RTs increase in face recognition when stimuli are inverted, confirming the difficulty of recognizing faces in this orientation (e.g., Itier and Taylor, 2002). However, we found this effect only for famous and unknown faces: response times for inverted familiar faces were not significantly higher compared to the same stimuli in upright condition. Some authors claim that the holistic processing used for upright faces is lost with inversion, and inverted faces, like objects, are processed only on the basis of their parts (i.e., Farah et al., 1995a,b). However, most of the studies that tested the effect of inversion have examined face recognition of famous and unknown people only (e.g., Itier and Taylor, 2002; Megreya and Burton, 2006). Hence, absence in literature of similar tasks makes it difficult to provide an exhaustive explanation of the phenomenon. We can suppose the holistic configuration is less compromised in inverted condition in function of familiarity. Further research is however needed.

Regarding the third issue, interaction between familiarity, expression and orientation in upright condition, our data show that subjects are faster in evaluating negative familiar faces than neutral ones. No differences were found across levels of expressions when famous and unknown faces were shown. Our results are consistent with studies that emphasize the joint effect of familiarity and expression in face recognition (Baudouin et al., 2000; Gallegos and Tranel, 2005; Dobel et al., 2008). One explanation for this pattern of results is based on the assumption that face recognition is easier if faces display typical rather than atypical expressions. So, there is a “*perceptual learning*” that defines the type of cognitive representation of known faces (Kaufmann and Schweinberger, 2004). It has been claimed that famous faces are depicted more frequently displaying one typical expression (generally positive) than all possible ones and resulted in faster recognition when smiling. Our famous stimuli varied for typical expressions (in the Italian media-context Vittorio Sgarbi is more frequently portrayed with negative expressions than positive expressions, unlike Roberto Benigni, while for other stimuli such as Queen Elizabeth or Barack Obama it is difficult to establish). This could partially explain the lack of differences in our results between expressions in this class of stimuli. Nevertheless, perceptual learning explanation cannot support our results for familiar faces. It is difficult to assume that there is a prototypical emotional representation for each family member, since the history of the relations are too varied to expose a subject to just one of their emotional expressions. And even if there were, it would be characterized by an extremely high inter-individual variability. One could argue that our subjects chose relatives with whom they had a higher affinity and good relationship and therefore cognitive representations of them were characterized by positive expressions. However, it is likely that the “expressive” representation of a relative is influenced by his character or his personality but plausibly independent from affection (for example if one has a taciturn or sulky disposition, his face representation will be characterized more by a neutral expression than positive, but this does not imply less affection towards him). So, regarding stimuli used in this experiment, the absence of a distinctive prototypical representation was, for different reasons, a common condition for both familiar and famous faces.

In regard to the inverted condition, some interesting results were obtained. Emotional expressions had an influence only on famous and unknown faces. No differences were found between the three expressions in the “familiar” condition. Literature regarding the processing of emotive expressions in inverted condition, is quite scarce and heterogeneous. While some studies show a detrimental effect of inversion on the recognition of all expressions, apart from positive ones (McKelvie, 1995; Calvo and Nummenmaa, 2008), some others reported an inversion effect for all types of emotions (Prkachin, 2003) or even opposite results, with happy faces being more affected by inversion than the others (Goren and Wilson, 2006). In our study, despite instructions not to explicitly recognize the emotions presented, our results seem to confirm those studies that show an easier processing of positive expressions also in inverted condition (Leppänen and Hietanen, 2004; Bombari et al., 2013). Again, familiar faces seem to constitute a distinct type of stimuli, being minimally affected by inversion in the analysis of the emotive effect.

### Philosophical phenomenological view

In division 1 of Being and Time, Heidegger (1996) argues that we ordinarily encounter objects as equipment, that is, as being for certain sorts of tasks (hammering, writing, etc.). He states that we do not generally encounter beings as detached, theoretical entities [*Vorhanden*] but as available or “ready-to-hand” [*Zuhanden*] and entwined in a tacit, holistic contexture of equipment (Ratcliffe, 2002). This account is reinforced by Merleau-Ponty (1962), who claims that the perceived object is always contextualized, not just by its physical surroundings, but by the particular projects and interests of the perceiver: the particular and potential actions that the perceiver is engaged in or could be engaged in. As Noë notes: “Perception is not something that happens to us, or in us. It is something we do […] What we perceive is determined by what we do (or what we know how to do); it is determined by what we are ready to do. In ways I try to make precise, we enact our perceptual experience; we act it out” (Noë, 2004). Hence, by following a phenomenological approach, perception is an active process that is structurally embodied and embedded, but it is possible to argue that the perception of a person is different when compared to the perception of objects. Recognizing a human face means to become aware of a particular kind of percept—the face of another human being like me—but it does not always mean to identify a “person”: a person is a human being regarded as an individual, an individual is a single human being as distinct from all other human beings (Liccione, 2013). Moreover, in encountering another person the most pressing task is relational engagement, and the way for which this engagement can be achieved depends upon many (inter)subjective and contextual factors, such as facial expressions. When we perceive an unknown person’s portrait we recognize a “face” (not an object), but our relational engagement with him/her is based only on the mere social meaning of his/her facial expression (i.e., in terms of approach/escape behavior). So, his/her identity and possible relational engagement are not interrelated. When we perceive a famous face, like that of Barack Obama, we really individualize a “person”—Barack Obama—the current president of the U.S., that is, a human individual with specified personality characteristics, so our relational engagement with him is based on our “media” knowledge. In this case, identity is an important factor but recognition of Barak Obama do not take the shape of a personal and unique historical pattern of relational engagements. Instead, recognition of our mother’s face occurs in a context of an exclusive and unique historical pattern of interactive opportunities that are so salient as to be constitutive of their recognition. Identity is a decisive factor.

Burton et al. (2005), proposed that the better recognition of familiar faces, with respect to unfamiliar ones, is due to a more functional refinement of stored representations of the former. This fine-tuning of representations of faces is “exposure-driven”, that is each new image of a face gradually upgrades its abstract representation, merging features that are constant across all possible variations. Our results (lower RTs for familiar faces) can well fit with this explanation given that it is possible argue that a person is more exposed to the faces of his/her family members than to those of celebrities and therefore he/she holds more powerful abstract representations of the former faces than those of the latter. Our familiar stimuli encompass several categories of relatives (e.g., parents, spouse, partner, etc.) for which it is likely to assume a different frequency of occurrence of encounters and, consequently, various refinement degrees of their abstract representations. Therefore, in order to verify frequency hypothesis it would be necessary provide experimental control of variables related to exposure effects (such as length of acquaintanceship with each relative and how long a subject spent time with him/her). In this way it would possible to examine if response times among familiar stimuli are or not affected by frequency of exposure. We cannot establish it solely with the data of this study.

Exposure time is often referred to domain of vision: Johnston and Edmonds (2009), correctly wrote that celebrities “may be very well known to the participants for a long period of time, *have been seen* in many different views and contexts, *have been seen* on many different occasions, and *have been seen* for lengthy periods of time (our italics)”. Let’s take a hypothetical example in which a family member and a celebrity have the same exposure time to the subject (i.e., a distant relative and a very famous anchorman). It is possible to argue that their cognitive representations share identical degrees of refinement and the same level of robustness to variation. Nevertheless, there is an important issue in supposing different qualitative aspects of quantitative exposure to these faces (famous and familiar targets): the celebrity’s face never “looked” at me, that is she never directed her gaze toward my person and, correspondingly, although I have *seen* his face, I have never *looked* at it. There is no real (eye) contact with famous faces since there is no intentional reciprocity for engagement. According to Stawarska (2006), mutual gaze implies an attention contact, yielding social attunement: intentional gaze toward the eyes of another, returned by him, allows for a second-person relation while observations without contacts produce a third-person relation. Cole (1999), claimed that in our social relationship we “exchange or share a mutual gaze”. Cooperative visual attention is a considered fundamental step for cognitive development and especially for social and emotional competences (Stawarska, 2006) and recently Mason et al. (2004) have shown that gaze direction contributes to the memorability of others. In our study, subjects were asked to produce a recognition judgment (whether the face was known or not) and familiar faces were the only targets for which it is possible to argue a past history of mutual glances. Moreover, the mutual gaze between members of the family is affectively characterized, unlike with strangers. We can argue that these qualitative aspects are doubtless unique for personally familiar faces, even if exposure is a decisive factor that strengthens familiarity (Burton et al., 2005), affective and emotional aspects related to personal narratives with others seem to play a special role in face processing. In their study (Gobbini et al., 2004; Gobbini and Haxby, 2007), showed different neuronal activation patterns in response to familiar faces, compared to famous or unknown ones, and these data are confirmed by other fMRI studies (Todorov et al., 2007; Vuilleumier and Pourtois, 2007). As argued by Gobbini et al. (2004) and Gobbini and Haxby (2007), interpersonal relationships towards familiar members provide a “person knowledge”, a set of salient biographical and autobiographical information that are *integral components of cognitive representation* of them (our italics).

According to this vision, it is possible to argue that the encounter with expressive famous faces does not have the same meaning as that connected to family members, that is the same quality of personal significance with relatives: expressions displayed on familiar faces are linked to memories that imply particular relational engagements and these can co-occur with recognition. Ratcliffe (2008), argues that “feelings of familiarity […] or relatedness […] can play a role in constituting the sense that a perceived entity is a remembered entity”. In other words, the relational horizons towards “my” mother (also) contribute to the recognition of her as “my” mother.

Arciero and Bondolfi (2009), claim that “at pre-reflective level, e-moting is the embodied meaning of an ongoing situation, perceived as a global mode of feeling and concurrently as a relational domain”. We can consider the “emotional face” as a salient cue of this relation domain that discloses new possibilities of action and passion. Indeed Cole (1999), argues that face-to-face encounters involve feeling toward and between people and that other faces put a “demand” on one, that is, it requires responding and entering into a relationship. So, expressive faces always imply my Self. Social meaning of facial expressions for Self is often singled out to explain different behavioral responses to angry and happy faces: positive expressions evoke approval and satisfaction with our conduct while angry expressions denote disconfirm (D’Argembeau et al., 2010). Both confirmation and disconfirmation of the Self move the subject to relational acts (to speak, to smile, to discuss, to embrace) and in this sense sad expressions elicit concern and call for caring. We suppose, however, that the relation between significance of expression and Self is (more) meaningful when it actually implies the Self. Instead, there are no angry, sad or happy faces but rather angry, sad or happy people with which the subject has different relational engagements. Consequently angry an expression by Barack Obama does not involve a sense of disconfirmation, as it would be as if the same expression were displayed on one’s mother’s face! The same can be said for sad and happy expressions. This can explain our results about interplay between identity and emotive expression and particularly results for the facilitation role of negative familiar faces. Indeed negative expressions are associated with “critical” relational contexts and can similarly imply negative emotional responses (such as concern, worry, quandary, but also sadness and anger): we can suppose that when these expressions are displayed by significant others the Self is more involved because of significant past relational engagement with them.

To summarize, famous and familiar faces are therefore different in respect to the historical conditions that have shaped and structured the experience with the person that these images depict. When we perceive faces, we are required to potentially actualize relational engaging, but if the faces carry an affective historical (past), engaging will be better recognized because historical relationships with them have the nature of *lived experiences*. Therefore, familiarity represents an indispensable condition for the perception of another’s face to be connected to a history of relational engagement. It is not a stimulus that is added to the perceptive structure of the face, but rather an embodied meaning which manifests itself in the face of a familiar person, inevitably referring back to the self. This phenomenological point of view explains why the holistic perception of a familiar face is maintained even if inverted: in our study, RTs for inverted faces showed no significant differences compared to those for upright faces. If we consider familiarity as constitutive to perception, and not only as perceptive content, it is therefore plausible that inversion can in no way act on it, unless the facial structure is so deformed as to render recognition impossible. In reference to this inverted condition, the results regarding the relationship between emotive expressions and class are questionable, for this reason further research is necessary to repeat the data.

### Limits

We used a limited set of faces that were repeatedly presented to subjects across upright and inverted condition. This may have resulted in a lower uncertainty during recognition tasks.

We conducted a pilot study to evaluate the recognition of positive, negative and neutral expressions displayed by famous faces. We did not plan a similar questionnaire addressed for assessing same expressions depicted by familiar faces.

We did not collect data for familiar faces regarding (1) length of acquaintanceship, (2) how long a subject spent time with them and (3) degree of appreciation for each familiar member. It is possible to argue that our concept of “familiarity” is independent (unrelated) to the first two variables (at least) but we cannot establish it solely with the data of this study.

The results of the present study showed that face recognition is facilitated by familiarity and emotional expression, emphasizing the distinction between famous and personally familiar faces and stressing importance of historical aspects from a phenomenological point of view.

## Conflict of interest statement

The authors declare that the research was conducted in the absence of any commercial or financial relationships that could be construed as a potential conflict of interest.

## Footnotes

^1^Roughly, defining perception as *embodied* means to consider the important role of the body shape in perceiving and experiencing the world and how we act in it. The idea that perception is *embedded* (and, on this ground, also “*extended*”) claims that perception is always situated in the environment: the objects (or events) are not isolated entities but instead, in Heidegger words, “at hand”, i.e., available to manipulation, and in this sense, they shape our perceptions and actions. *Enactive* dimension of perception reveals that it is not merely analysis of actual physical features of objects but perception calling for action. For more detailed explanations see Gallagher (2008) and Noë (2004).

^2^Here we use the word “sense” to explain the idea of direction, purpose, motivation etc.
